# Preference of individuals in the treatment strategies of acute myocardial infarction in China: a discrete choice experiment

**DOI:** 10.1186/s12955-020-01466-1

**Published:** 2020-07-07

**Authors:** Weiqian Dai, Chang Liu, Jiahe Liu, Yaduan Lin, Yu Cheng, Wai-kit Ming

**Affiliations:** 1 Department of Public Health and Preventive Medicine, School of Medicine, Jinan University, 601 Huangpu W Ave, TianHe, Guangzhou, Guangdong China; 2 Harvard Medical School, Harvard University, Boston, MA USA

**Keywords:** Myocardial infarction treatment, Choice behavior, Discrete choice experiment

## Abstract

**Background:**

Acute myocardial infarction (AMI) is a significant cause of mortality and morbidity worldwide. Today, with increasing life quality and social economy, people pay much attention to the cost-effectiveness of a treatment strategy. This study investigated the preferences of individuals who would be potential caregivers or patients for AMI treatment in order to provide liable and instructive information for cardiologists and other related physicians.

**Methods:**

A discrete choice experiment was conducted among people to assess preferences for hypothetical AMI treatment scenarios characterized by the attributes of treatment method, mortality within 5 years, complication rate within 1 year, treatment duration and expense. A conditional logit regression model and latent class analysis were used to interpret the collected data systematically. The relative importance of each attribute and willingness to pay of people on the trade-offs between different treatment strategies were estimated.

**Results:**

Participants valued mortality within 5 years most highly (average importance: 40.9, 95%CI 0.447–0.530). Three classes of participants were identified: Class 1 placed the most importance on treatment duration, class 2 corresponded with the overall result while expense was regarded as the most important attribute in class 3. Individuals favored an intermediate treatment duration of about 10 days instead of the shortest (95% CI 1.044–1.248, *P* < 0.001). People’s characteristics (sex, age, marriage, education and income) affected their preferences (*P* < 0.01).

**Conclusion:**

People considered a mortality rate within 5 years as the most crucial attribute in the MI treatment and preferred an intermediate treatment duration of about 10 days. Furthermore, the findings estimated the trade-offs acceptable to patients and heterogeneity in preferences for AMI treatment.

## Background

Acute myocardial infarction remains the leading cause of morbidity and mortality in both men and women around the world [1]. It occurs more frequently in adults over 40 years old, and morbidity and mortality will increase with age. Recently, the average age of AMI onset tends to be younger [2]. Everyone is at risk of AMI, especially those with risk factors such as smoking, obesity, and diabetes. Also, many will become caregivers for family members affected by AMI [3]. When people are sick, most of them will discuss with or inquire about their family members such as parents, spouses, children, or other relatives about their health situation and advice on treatment. Those people who are caregivers might play an important role in the decision-making process. With the quality of life increasing and medical techniques improving, people pay greater attention to the effectiveness and economic efficiency of a treatment strategy. Age, income levels, educational backgrounds, and other sociodemographic characteristics are contributing factors in people’s choices. However, the preferences of individuals, especially of potential caregivers about AMI treatment, are unclear. As a result, it is critical to investigate the preferences of individuals to better understand what people attach most importance to when they are helping families or themselves make a choice [4].

Discrete choice experiment (DCE) is a useful technique to elicit patients’ relative preference weights for harmful or beneficial treatment outcomes. These weights reflect the trade-offs that an individual is willing to make among different treatment outcomes when choosing among treatment options, which are described by different attributes [5]. In this article, we will explore the homogeneity and heterogeneity in the preferences of people using conditional logit and latent class analysis, respectively. Conditional logit is one of the most widely used methods to analyze data from the similarity in health economics. Latent class analysis groups respondents into a pre-specified number with distinct preferences. This allows for the estimation of class-specific preferences [6].

Nowadays, the attitudes of people about seeing a doctor have changed, and most of them are more willing than ever before to participate in the medical decision-making process. However, doctors might not have sufficient knowledge regarding the actual intentions of people, especially caregivers. Therefore, this study aims to investigate the preference of people aged 20--50 years who are potential AMI targets or caregivers in the treatment of myocardial infarction. The results should provide many useful guidelines for doctors to conduct suitable treatment plans for each patient These findings can also be helpful in reducing treatment time, costs, and use of medical resources.

## Methods

### Study design and procedure

Current guidelines [7, 8] for conducting DCEs are the basis for the study design and analysis. The DCE methodology is grounded in multi-attribute utility theory in economics and is a way of measuring individuals’ valuation of different aspects of healthcare technologies [9]. This theory assumes that any commodity can be characterized by severe key attributes and levels (e.g., treatment therapy, duration, and expenses), and that people make choices among these options by comparing the attributes and levels.

Therefore, the first step to designing a questionnaire is to select suitable attributes and levels. An extensive literature search led to the selection of the characteristics of treatment for myocardial infarction. Five attributes were chosen: therapies for myocardial infarction, mortality within 5 years, treatment duration, complication rate within 1 year, and expenses. We chose these five attributes according to the frequency of those factors in the literature, which were very high, to be used to describe the treatment of AMI. Those attributes described the feature of the treatment of AMI, including methods, costs, and outcomes. We discussed the provisional attributes with cardiologists at Jinan University Affiliated Hospital, and they considered these factors valid and feasible. Levels of therapies for myocardial infarction were determined according to the 2016 AHA guidelines on acute myocardial infarction [10]. This study selected minimum and maximum levels of other attributes from related reports [11–13] and experts’ opinions in order to cover all possibilities of people’s choices, and intermediate levels were calculated by a median value between minimum and maximum. The details are presented in Additional File 1.

There were two parts in this questionnaire. The first part sought to collect sociodemographic information from participants, including age, educational background, and annual income (see Additional File 2). The second part contained seven questions to describe a hypothetical situation in which a person was at an increased risk of having an acute myocardial infarction, and they had to make a choice among different treatment options. Each question included three options: option A, option B, and “None”. For each question, participants were required to choose one of the three options that they perceived to be better by comparing their attributes. The next six-choice questions followed a similar format, but test profiles varied as we changed the attribute levels in each question each time and asked the participants to make their choices based on the new test profiles. Using this approach, we can understand the impact of test attributes on the choices made. To ensure that respondents could understand our questionnaire, we also provide notes on the purpose of this study and a detailed explanation of each attribute and terminology. Besides, there were special officers responsible for questionnaire distribution, if the respondents were confused about the questions, they could contact us, and we would answer their questions (the website of the questionnaire and detailed explanation on each attribute are seen in Additional File 3).

Theoretically, a large number of hypothetical options (about 405) can be generated through various combinations of attributes and their levels. Presenting all possible choice questions in a DCE is not practical. Therefore, we used a fractional factorial design [14] in which we selected a subsample of 180 questions that were grouped into seven versions of the DCE questionnaire. Sawtooth software was used to create the fractional factorial design that met balance and orthogonality properties [7]. This design ensures that each attribute level appears equally often within an attribute (balance), and each pair of levels appears equally often across all pairs of attributes (orthogonality), which minimizes the bias and improves the precision of estimated preferences. A total of 100 versions of the questionnaire were generated, and each respondent was randomly assigned to a version to facilitate balance and orthogonality. The seven questions were all randomly chosen. This is a web-based questionnaire that facilitated direct data entry into our secure server, and the questionnaire was conducted using the Choice Based Conjoint application of Sawtooth (SSI Web version 9.4.0; Sawtooth Software Inc).

### Study sample

Our questionnaire was aimed at the entire population in mainland China, Hong Kong and Macau. The questionnaires were presented online through Wechat Moments, Weibo and other social network platforms. By these means, every respondent answered the questions voluntarily, and the information from people with all different backgrounds could be collected. This research was carried out between October 2017 and September 2018.

### Statistical analysis

A conditional logit regression model and latent class analysis (LCA) were used to interpret the collected data. The conditional logit regression model was used to quantify the correlation between the choice made and the attribute levels of various test profiles where the choices were used as the dependent variable and the attribute levels of the tests were used as covariates [15]. The conditional logistic model provided statistical inferences about respondents’ preference weights for each of the attributes and levels included in the questionnaire. Positive or negative coefficients generated by the regression analysis indicated the direction of the preference for each attribute. The significance and size of coefficients could also estimate the relative importance of the attributes. We calculated the marginal rates of substitution (MRS)(i.e., the ratio of two coefficients, to measure the willingness to accept a trade-off among different options). These MRS values allowed for the comparison of different attributes using a common scale [16].

The latent class analysis was conducted to identify classes of individuals with similarities in their preferences [17–19]. It used a semiparametric approach to model the correlation structure of the data and identified classes that are more homogeneous in terms of variance structure. As a result, we could identify distinct classes of the population in our sample in terms of their preference patterns using this approach. Based on Akaike Information Criterion 3 (AIC3) and supported by Bayesian Information Criteria (BIC), the optimal number of classes was determined in an iterative procedure, by making comparisons of models with different numbers of classes [20]. Willingness to pay for each attribute was calculated using results from the most appropriate regression model that described how much people were willing to pay to avoid something or choose better treatment (details of the calculating model was presented in Additional File 4). This technology can help us to understand possible heterogeneities among the participants’ preferences. All statistical analyses were performed using Sawtooth Lighthouse Studio (SSI Web version 9.4.0; Sawtooth Software Inc).

## Results

A total of 383 people who participated in this survey and provided complete responses to all questions were included in the analysis. Initially, 454 participants were approached during the collection process; of those, 71 people (15.6%) did not complete all of the questions and were excluded from the analysis.

The mean age in our sample was 30.4 years; among these, 42.8% were male and 57.2% were female. Most participants had relatively high levels of education (74% were graduates or had higher education). The percentage of people or their family members who had heart diseases previously was 62.6%. Meanwhile, the health examination rate was relatively low, accounting for 35.6%. The demographic information of the participants is summarized in Additional File 5.

### Overall preference and willingness to pay

**Fig. 1:**
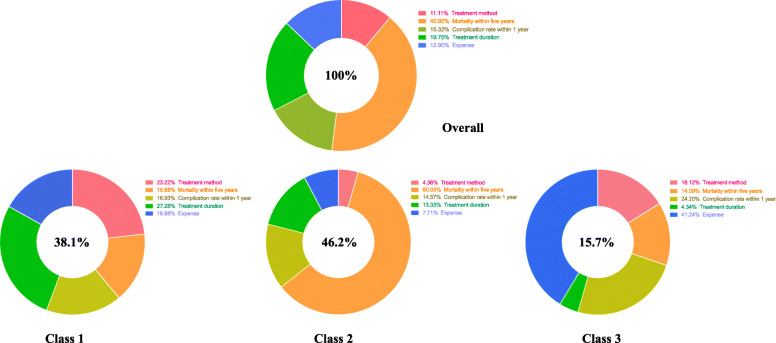
Importance of attributes for overall and by class. Overall. The red area means importance of attribute of treatment method is 11.11%. The orange area means importance of attribute of mortality within five years is 40.92%. The grass-green area means importance of attribute of complication rate within one year is 15.32%. The bright-green area means importance of attribute of treatment duration is 19.75%. The blue area means importance of attribute of expense is 12.90%. 11.11% Treatment method.  40.92% Mortality within five years.  15.32% Complication rate within 1 year.  19.75% Treatment duration.  12.90% Expense. Class1. The red area means importance of attribute of treatment method is 23.22%. The orange area means importance of attribute of mortality within five years is 15.68%. The grass-green area means importance of attribute of complication rate within one year is 16.93%. The bright-green area means importance of attribute of treatment duration is 27.28%. The blue area means importance of attribute of expense is 16.88%.  23.22% Treatment method.  15.68% Mortality within five years.  16.93% Complication rate within 1 year.  27.28% Treatment duration.  16.88% Expense. Class 2. The red area means importance of attribute of treatment method is 4.36%. The orange area means importance of attribute of mortality within five years is 60.03%. The grass-green area means importance of attribute of complication rate within one year is 14.57%. The bright-green area means importance of attribute of treatment duration is 13.33%. The blue area means importance of attribute of expense is 7.71%.  4.36% Treatment method.  60.03% Mortality within five years.  14.57% Complication rate within 1 year.  13.33% Treatment duration.  7.71% Expense. Class 3. The red area means importance of attribute of treatment method is 16.12%. The orange area means importance of attribute of mortality within five years is 14.09%. The grass-green area means importance of attribute of complication rate within one year is 24.20%. The bright-green area means importance of attribute of treatment duration is 4.34%. The blue area means importance of attribute of expense is 41.24%.  16.12% Treatment method.  14.09% Mortality within five years.  24.20% Complication rate within 1 year.  4.34% Treatment duration.  41.24% Expense

**Fig. 2:**
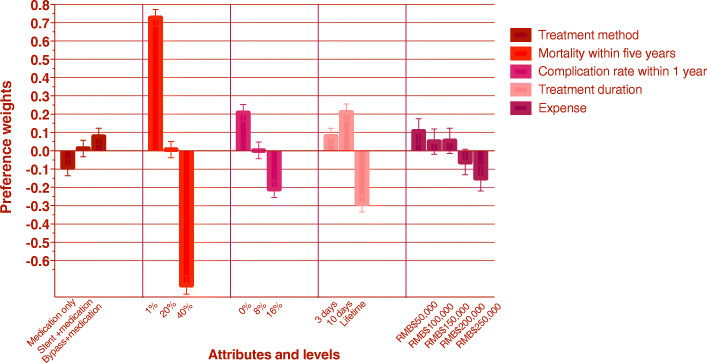
The visual presentation of estimated overall preference weight. The dark red bar(first group ) indicates the preference weight of treatment method. The bright red bar(second group ) indicates the preference weight of mortality rate within five years. The light purple bar(third group ) indicates the preference weight of complication rate within one year. The pink bar(fourth group ) indicates the preference weight of treatment duration. The dark purple bar(last group ) indicates the preference weight of expense. Treatment method. Mortality within five years. Complication rate within 1 year. Treatment duration. Expense

Overall, the respondents in our study considered mortality rate within 5 years to be the most important attribute, followed by treatment duration, complication rate within 1 year and expense. Treatment methods were regarded as the least important aspect of the treatment course (Fig. 1). The results of the conditional logit model are presented in Table 1, which includes estimated average preference weights, standard error, and *p*-values for all attribute levels. The estimated odds ratios and their confidence intervals are also reported in Table 1. Figure 2 provides a visual presentation of the estimated preference weights in our study sample (*n* = 383). Participants reported not wanting to take medications but preferred to undergo bypass surgery rather than stent intervention. They gave a negative preference weight for the higher mortality rate within 5 years. Using a 1% mortality rate within 5 years as a reference group, the odds ratio of choosing 20% would be 0.487 (95%CI 0.447–0.530), and the odds ratio of choosing 40% would be 0.232 (95%CI 0.211–0.255). The negative preference weights increased with the increasing complication rate within 1 year. The odds ratios of choosing 8 and 16% complication rates were 0.814 (95%CI 0.745–0.890) and 0.659 (95%CI 0.602–0.721), respectively. The ten-day duration was highly preferred by participants. The estimated preference weight for 10 days was positive; and for a lifetime, it was negative (*P* < 0.001). The odds ratio of opting for a 10-day duration would be 1.142 (95% CI 1.044–1.248); for a lifetime duration, it was 0.693 (95% CI 0.633–0.759). The estimated preference weights were positive for treatment expenses ranging from RMB$ 50,000 to RMB$ 150,000, and negative for those exceeding RMB$ 150,000; though both tended to increase. The odds ratios of choosing RMB$100,000, RMB$150,000, RMB$200,000 and RMB$250,000 were 0.946 (95%CI 0.826–1.083), 0.949 (95%CI 0.829–1.086), 0.846 (95%CI 0.738–0.969), and 0.774 (95%CI 0.675–0.888) respectively. The coefficient signs of the mortality rate within 5 years, complication rate, and expenses suggest that people prefer a treatment method with a lower mortality rate, lower complication rate, and less cost.

**Table 1:** Estimated Relative Preference Weights for overall

	Estimated preference weights				Odds ratio	
Attributes	Level	Coefficient	Standard Error	*P* value	Odds ratio	95% CI
Treatment method	Only medication	−0.090	0.046	0.048	reference	
	Stent intervention + medication maintenance	0.013	0.046	0.780	1.109	(1.014–1.212)
	Bypass surgery + medication maintenance	0.078	0.045	0.088	1.183	(1.082–1.293)
Mortality within five years	1%	0.727	0.046	< 0.001	reference	
	20%	0.007	0.044	0.875	0.487	(0.447–0.530)
	40%	−0.734	0.049	< 0.001	0.232	(0.211–0.255)
Complication rate within one year	0%	0.208	0.045	< 0.001	reference	
	8%	0.002	0.045	0.969	0.814	(0.745–0.890)
	16%	−0.209	0.046	< 0.001	0.659	(0.602–0.721)
Treatment duration	3 days	0.078	0.045	0.084	reference	
	10 days	0.211	0.045	< 0.001	1.142	(1.044–1.248)
	lifetime	−0.289	0.047	< 0.001	0.693	(0.633–0.759)
Expense	RMB$50000	0.106	0.069	0.124	reference	
	RMB$100000	0.050	0.069	0.466	0.946	(0.826–1.083)
	RMB$150000	0.054	0.069	0.433	0.949	(0.829–1.086)
	RMB$200000	−0.061	0.069	0.378	0.846	(0.738–0.969)
	RMB$250000	−0.149	0.070	0.033	0.774	(0.675–0.888)
Log-likelihood		− 1313				
Log likelihood of model without predictors		− 1950				
Akaike Info Criterion (AIC)		2708				
Bayesian Information Criterion (BIC)		2933

Participants had a strong preference for the mortality rate within 5 years and were willing to pay up to RMB$29,291 (approximately US$4323) for a 0% mortality rate. Also, people were willing to pay RMB$20,373 (US$3007) to reduce the complication rate by 1% and up to RMB$14,789 (US$2183) to increase the 3-day duration to a 10-day duration. People were less sensitive to treatment methods, and they were willing to pay RMB$9, 964 (US$1471) for bypass surgery (Table 2).

**Table 2:** Willingness to pay

Attribute		Willingness to pay							
		Overall N = 383 (100%)		Class 1 *n* = 146 (38.1%)		Class 2 *n* = 177 (46.2%)		Class 3 *n* = 60 (15.7%)	
		RMB ($)	USD ($)	RMB ($)	USD ($)	RMB ($)	USD ($)	RMB ($)	USD ($)
Treatment method	Stent intervention + medication	60,748	8966	706,776	104,319	71,878	10,609	78,191	11,541
	Bypass surgery + medication	9964	1471	573,881	84,705	58,197	8590	23,280	3436
Mortality within five years		29,291	4323	34,417	5080	45,931	6780	1463	216
Complication rate within one year		20,373	3007	90,535	13,363	27,166	4010	6562	968
Treatment duration	10 days	14,789	2183	67,716	9995	13,497	1992	3008	444
	lifetime	20,478	3023	13.2909	19,617	21,667	3198	961	142
Expense		Reference		Reference		Reference		Reference	

$1 RMB = $0.15USD

### The preferences and willingness of the three classes to pay

The AIC and BIC were minimized for the LCA models with three classes, suggesting three segments of participants were present in the data. The preference weights for test attributes are presented in Fig. 1 for the three classes: 146 (38.1%), 177 (46.2%), and 60 (15.7%) fell into class 1, class 2, and class 3, respectively. Class 1 had similar attribute-importance preferences toward each attribute; they had relatively more substantial attribute importance toward treatment duration (27.28%) and treatment methods (23.22%). Class 2 was more sensitive to the mortality rate within 5 years, and class 3 attributed high importance to treatment expenses. The preference for complication rate within 1 year varied and was relatively unimportant among the three groups.

**Fig. 3:**
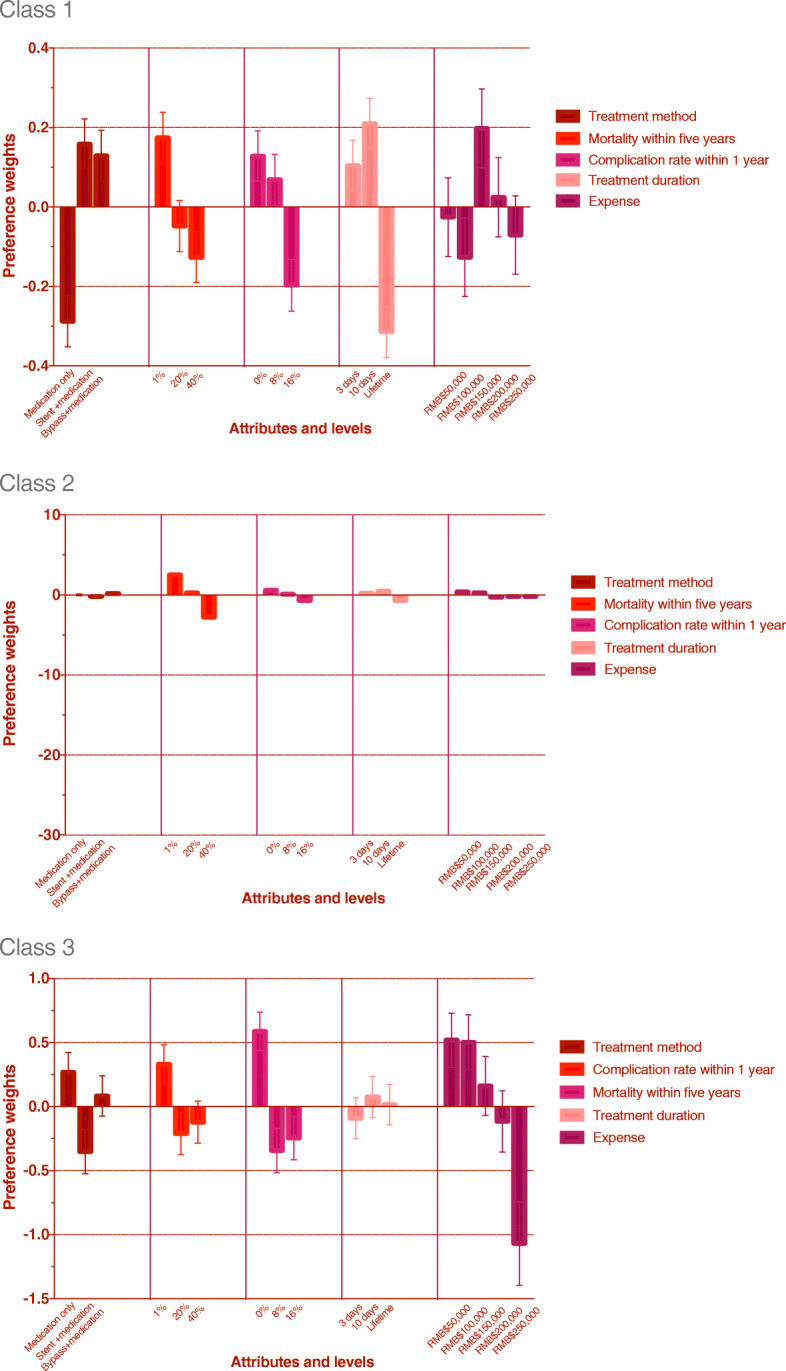
Tthe visual presentation of estimated preference weight for three classes. The dark red bar(first group ) indicates the preference weight of treatment method. The bright red bar(second group ) indicates the preference weight of mortality rate within five years. The light purple bar(third group ) indicates the preference weight of complication rate within one year. The pink bar(fourth group ) indicates the preference weight of treatment duration. The dark purple bar(last group ) indicates the preference weight of expense. Treatment method. Mortality within five years. Complication rate within 1 year. Treatment duration. Expense

The results of the conditional logit model for three classes, including estimated average preference weights, standard error, and *p*-values for all attribute levels, are shown in Table 3. A visual presentation of the estimated preference weights is displayed in Fig. 3. For class 1, people preferred stent intervention over the other two methods. An 8% complication rate can be acceptable, and a 10-day treatment duration is the first choice for those people. In contrast to common sense, where we might believe that people will opt for the least expensive method, in class 1, people preferred the RMB$150,000 treatment strategy. In class 2, people preferred to undertake bypass surgery and the 10-day treatment duration. The complication and mortality rates are also important concerns. Participants in class 3 are more sensitive to the complication rate than the other two groups. Medication, low mortality rate, and 10-day duration are preferable, and people are not willing to pay more than RMB$150,000 for treatment.

**Table 3:** Estimated Relative Preference Weights for three classes

Attribute		Class 1 n = 146 (38.1%)			Class 2 n = 177 (46.2%)			Class 3 n = 60 (15.7%)		
		Coefficient	SE	*P* value	Coefficient	SE	*P* value	Coefficient	SE	*P* value
Treatment method	Only medication	−0.287	0.064	< 0.001	0.013	0.111	0.904	0.268	0.154	0.087
	Stent intervention + medication maintenance	0.159	0.064	0.014	−0.198	0.114	0.084	− 0.352	0.171	0.044
	Bypass surgery + medication maintenance	0.129	0.064	0.047	0.184	0.118	0.119	0.084	0.156	0.595
Mortality within five years	1%	0.175	0.064	0.007	2.509	0.166	< 0.001	0.332	0.152	0.032
	20%	−0.048	0.064	0.456	0.246	0.104	0.020	−0.211	0.166	0.209
	40%	−0.127	0.064	0.049	−2.755	0.192	< 0.001	−0.121	0.164	0.464
Complication rate within one year	0%	0.128	0.063	0.045	0.589	0.125	< 0.001	0.588	0.149	< 0.001
	8%	0.069	0.064	0.286	0.099	0.116	0.394	−0.343	0.173	0.052
	16%	−0.197	0.065	0.003	−0.688	0.125	< 0.001	−0.245	0.170	0.156
Treatment duration	3 days	0.104	0.064	0.109	0.205	0.113	0.073	−0.091	0.160	0.571
	10 days	0.210	0.064	0.001	0.482	0.119	< 0.001	0.076	0.159	0.635
	lifetime	−0.314	0.065	< 0.001	− 0.687	0.123	< 0.001	0.016	0.157	0.922
Expense	RMB$50000	−0.026	0.099	0.797	0.399	0.179	0.027	0.519	0.212	0.017
	RMB$100000	−0.126	0.099	0.204	0.277	0.176	0.119	0.502	0.214	0.022
	RMB$150000	0.198	0.100	0.049	−0.277	0.171	0.108	0.162	0.230	0.483
	RMB$200000	0.025	0.099	0.805	−0.210	0.167	0.209	−0.115	0.240	0.632
	RMB$250000	−0.070	0.099	0.477	−0.189	0.177	0.289	−1.068	0.327	0.002

As to willingness to pay (Table 2), people in class 1 were willing to pay RMB$706,766 (US$104,319) for stent intervention and RMB$573,881 (approximately US$84,705) for bypass surgery instead of taking drugs and were more sensitive to treatment duration than those in the other two classes, in which people were willing to pay RMB$67,716 (US$9995) for a 10-day treatment instead of a 3-day treatment but were willing to pay RMB$132,909 (US$19,617) to avoid lifetime treatment.

Participants in class 2 had a strong preference for a low mortality rate within 5 years and were willing to pay up to RMB$45,931(USD6,780) to reduce the mortality rate by 1%, assuming everything else was equal. People were willing to pay RMB$71,878 (USD10,608) to avoid stent intervention and RMB$58,197 (USD8,590) for bypass surgery. For class 2, people were also sensitive to the complication rate and were willing to pay RMB$27,166 (USD4,010) to reduce the complication rate by 1%.

Participants in class 3 had the lowerest importance score for a mortality rate within 5 years and were willing to pay RMB$1416 (USD216) for that. In contrast, they were willing to pay RMB$6562 (USD968) to reduce the complication rate within 1 year by 1%. Similarly, people were willing to pay RMB$78,191 (USD11,541) and RMB$23,280 (USD3,436) to avoid stent intervention and bypass surgery, respectively.

## Discussion

A discrete choice experiment was conducted to elicit the relative preferences of individuals regarding different attributes of the treatment plan. We found that when making choices among different myocardial infarction treatment options, people pay much more attention to a complication rate within 5 years than other attributes and are not willing to use only drugs to treat the infarction perhaps because drugs take a long time and they do not think drugs can completely cure the diseases. Bypass surgery is more favored by adults than stent intervention, while stent intervention is safer and more cost-effective than bypass surgery. Another survey that was conducted to investigate why these people preferred bypass surgery rather than stent intervention indicated that their seemingly illogical choices might be due to inadequate knowledge about stent intervention. Education on myocardial infarction should be improved so that people can make more rational decisions. Another interesting result is that people think a 10-day treatment is better than a 3-day treatment, which means they might believe the best treatment is not the fastest. A too short duration might make people feel the treatment is not reliable and effective. Treatment expenses within RMB$150,000 can be acceptable to most of the participants, which corresponds to their annual income. The treatment plan with the lower mortality rate, lower complication rate, and reasonable duration (about 10 days) and expenses are strongly preferred by adults.

In our study, the LCA revealed three classes of participants who had different preference patterns for various test attributes. Most people wanted more information about the treatment plan, and many wanted to increase their level of involvement in the decision-making process. A large part of the population, however, was unware of the inherent uncertainty in outcomes and the variation in performance and risks of the different treatment strategies. Guidelines for the choice of treatment scheme should consider people’s preferences, including that of their caregivers, and fulfill both patients’ needs and values, as well as the public health aspect of those choices. Therefore, there are difficult but inevitable choices that need to be made daily in clinical practice by physicians, especially for people with high risks for developing myocardial infarction. Additional knowledge about preferences, priorities, and concerns of the patient and their caregivers can help physicians in a shared decision-making process. This information might help physicians focus on the factors that are important for patients and their family and can influence their choice when deciding on a treatment plan.

The LCA has the advantage of exploring the association between preferences (class membership) and background characteristics that could explain whether sex, age, income or other factors affected their choices and how these factors influenced them (details seen in the Additional File 6). In our study, we found that age has a great impact on people’s choice of treatment therapy; older people are less willing to undertake stent intervention or bypass surgery and tend to pay more attention to complication rates. The effect of marriage is similar to that of age, which is puzzling and requires further study to determine the exact relationships. People with higher education tend to have a more careful consideration of the pros and cons of a treatment plan. Participants who have a high annual income are more attentive to treatment costs, while those with less income are not that sensitive to cost. This phenomenon could be explained by the fact that although people with higher annual income could afford the treatment of AMI, most of the participants in our study are exactly middle-aged and have the heaviest burden of life such as child-rearing, a house or car loan, and supporting the elderly. As a result, they would be more sensitive to cost. In China, people with lower incomes uauslly depend on social insurance and government medical insurance to pay for treatment, and they must pay only a little themselves. However, when the cost becomes higher and exceeds the range of insurance, most of would forgo the treatment because they could not afford it. In other words, it is not meaningful for them to further consider the costs because they could not afford the treatment. Our results suggest that clinicians might need to focus on the patients and their caregivers’ needs to improve the quality of the consultation process. Clinicians can use this information to provide more targeted consultations and concentrate on the risks and benefits of the test that might influence the final decision. This will ideally result in better choices since it incorporates the patient and their family’s concerns and preferences and the evidence on available diagnostic options in the decision-making process.

Our study has a limitation regarding the representativeness of our sample, which had selection bias because an electronic questionnaire was used, which would exclude segments of the population unaccustomed to the use of social networks due to their age, culture, or economic characteristics. Response bias is also a limitation of survey-based research; participants would respond to questions untruthfully or emotionally due to their interest in this subject or to social pressure among other reasons. Likewise, the sample size of participants aged between 30 and 50 years old was small.

Larger sample sizes and diversified methods of distribution have helped obtain more robust findings in the LCA analysis. Some inconsistencies in the order of estimated preferences that we have observed in our data might be a result of small class sizes, especially in class 3. Also, we only included five attributes to characterize myocardial infarction treatment. This was to avoid the complexity of choice questions and reduce the burden of questionnaires for the respondents. However, we acknowledge that choices regarding treatment might be affected by other factors such as the effectiveness of treatment therapy and the expertise of the physicians or surgeons. The inaccurate or limited knowledge of participants would also affect the results. To reduce these effects, we provided an introduction to the purpose of this research, basic information about acute myocardial infarction, the meaning of several terminologies, and a detailed explanation of each attribute and level. When faced with a real-life situation rather than a hypothetical one, people might opt for a different course.

In the future, an in-depth exploration of the processes that leads to decisions about myocardial infarction using qualitative methods can be conducted to inform the selection of attributes and the choice of questions.

## Conclusion

Decisions for myocardial infarction treatment depend on multiple factors, such as treatment methods, mortality rate, duration, and costs, and both patients and their caregivers’ preferences need to be considered to ensure their needs and preferences are met as much as possible.

In conclusion, people’s preferences for treatment methods indicate the need for effective treatment counseling and for ensuring people’s better understanding of the treatment plan. Also, people are concerned about treatment duration when making decisions regarding the treatment plan during which people do not think that the shortest treatment duration is the best. For young adults, the cost is not a determining factor for acceptability when the expense is around RMB$150,000. We identified classes of people who had different preferences for the diagnostic test attributes. Some of these differences in preferences could be partially explained based on age, marriage, education, and income. Our results might influence clinicians’ perceptions of the aspects of the treatment plan that need to be discussed with patients and their families during the consultation. Future studies conducted in larger and more representative samples are needed to enforce our current findings and to facilitate the measurement of potential preference heterogeneity among people.

## Supplementary information

**Additional file 1.** Attributes and levels for treatment options in the questionnaire.

**Additional file 2.** Baseline questions for the population in this study.

**Additional file 3.** The website of the questionnaire: http://t.cn/ROlvKtL.

**Additional file 4.**

**Additional file 5: Table 2**. Demographics and Characteristics of Patients in this Study.

**Additional file 6.** Demographics and characteristics of subgroups in this study.

## Data Availability

The datasets collected and/or analyzed during the current study are available from the corresponding author upon reasonable request.

## Abbreviations

AMI: Acute myocardial infarction
DCE: Discrete choice experiment
LCA: Latent class analysis
AIC3: Akaike Information Criterion 3
BIC: Bayesian Information Criteria
