# Supplementary material for: Preference of individuals in the treatment strategies of acute myocardial infarction in China: a discrete choice experiment
Source: Health Qual Life Outcomes. 2020 Jul 7;18:217. doi: 10.1186/s12955-020-01466-1 (PMC7339539; doi:10.1186/s12955-020-01466-1)
Supplement: Supplementary file 1 — Additional file 1. Attributes and levels for treatment options in the questionnaire. [file 12955_2020_1466_MOESM1_ESM.docx]

**Additional File 1------ Attributes and levels for treatment options in the questionnaire**

| Treatment attributes | Levels of attributes(regression coding) | |
| --- | --- | --- |
| Therapies for myocardial infarction | L1 | Only medication |
|  | L2 | Stent intervention + medication maintenance |
|  | L3 | Bypass surgery + medication maintenance |
| Mortality within five years | L1 | 1% |
|  | L2 | 20% |
|  | L3 | 40% |
| Complication rate within one year | L1 | 0% |
|  | L2 | 8% |
|  | L3 | 16% |
| Treatment duration | L1 | 3 days |
|  | L2 | 10 days |
|  | L3 | Lifetime |
| Expenses | L1 | 50,000 RMB |
|  | L2 | 100,000 RMB |
|  | L3 | 150,000 RMB |
|  | L4 | 200,000 RMB |
|  | L5 | 250,000 RMB |
