# Supplementary material for: Preference of individuals in the treatment strategies of acute myocardial infarction in China: a discrete choice experiment
Source: Health Qual Life Outcomes. 2020 Jul 7;18:217. doi: 10.1186/s12955-020-01466-1 (PMC7339539; doi:10.1186/s12955-020-01466-1)
Supplement: Supplementary file 2 — Additional file 2. Baseline questions for the population in this study. [file 12955_2020_1466_MOESM2_ESM.docx]

**Additional File 2 ------ Baseline questions for the population in this study**

| Sex: male/female |
| --- |
| Age |
| Marriage: unmarried/married/divorced/widow or widower |
| Education: primary/secondary/high school/graduate/undergraduate/PHD |
| Annual income |
| Whether did you or your family members have any heart diseases in the past? Yes/No |
| If there was, which kind therapy did you or your family members take? Medication/Stent/Bypass/others |
| Did you and your family members take a body check every year? Yes/No |
