# Supplementary material for: Preference of individuals in the treatment strategies of acute myocardial infarction in China: a discrete choice experiment
Source: Health Qual Life Outcomes. 2020 Jul 7;18:217. doi: 10.1186/s12955-020-01466-1 (PMC7339539; doi:10.1186/s12955-020-01466-1)

**Additional File 3**

**The website of the questionnaire:** <http://t.cn/ROlvKtL>


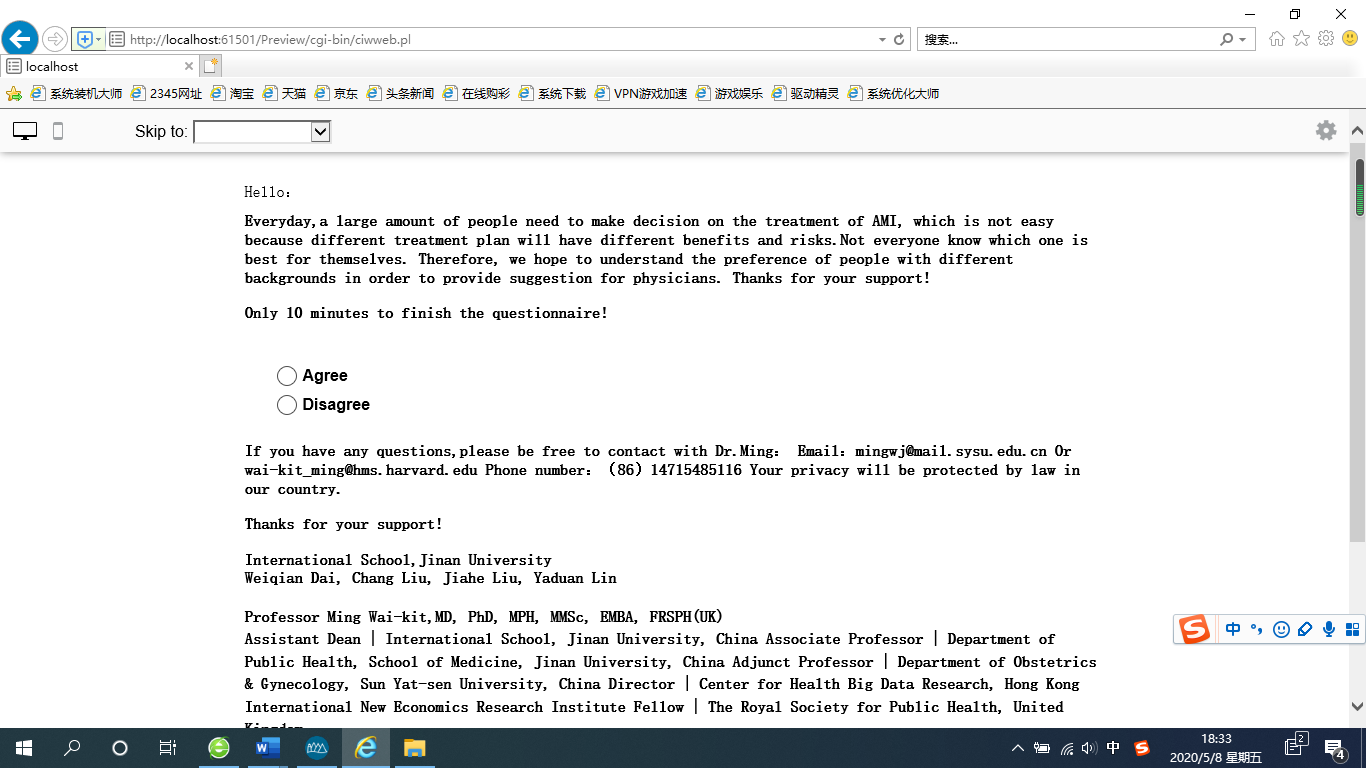

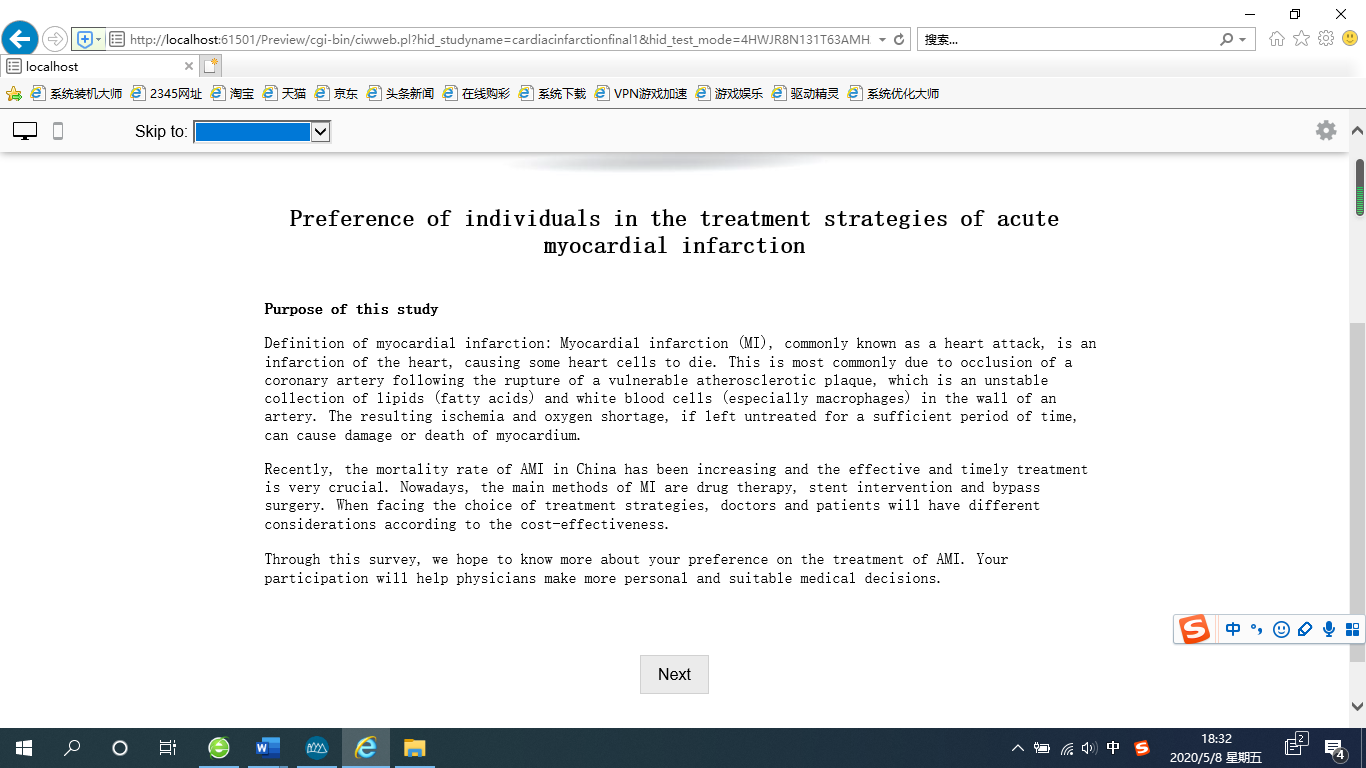
**Because the questionnaire is only Chinese, here we provide a translation version of each page of the questionnaire for the understanding of readers of our article.**


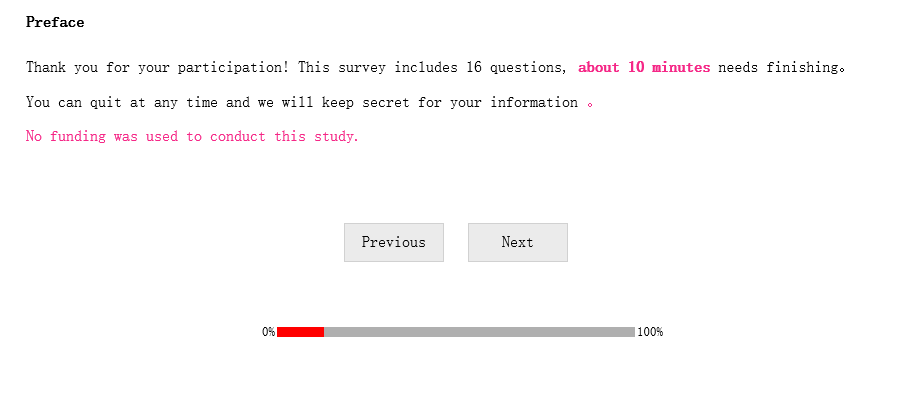


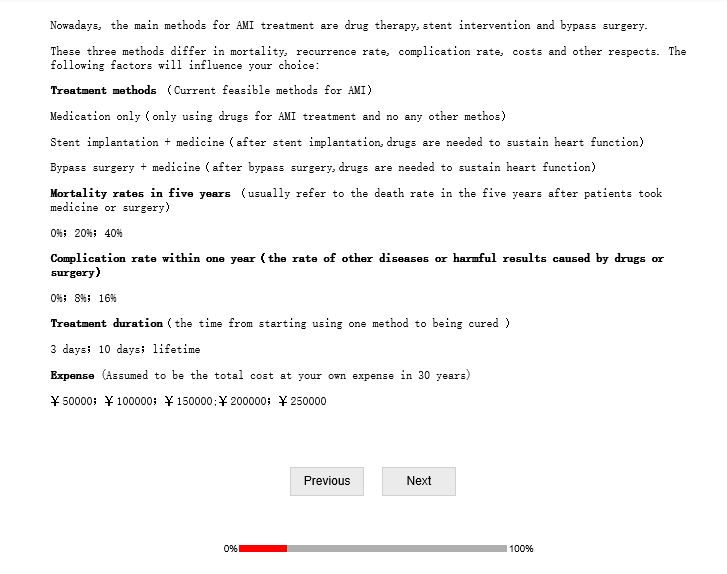


This page gives the detailed explanation on what each attribute and level mean to help participants to understand those choices.


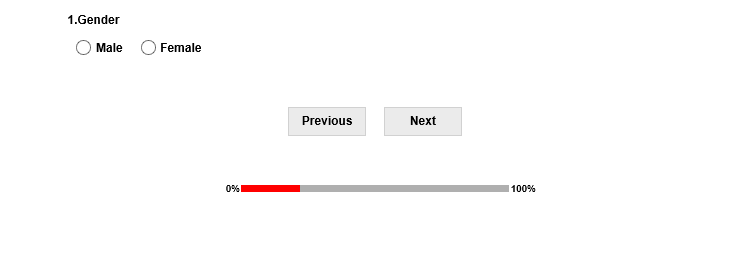


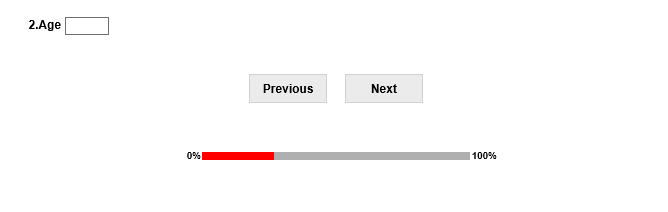


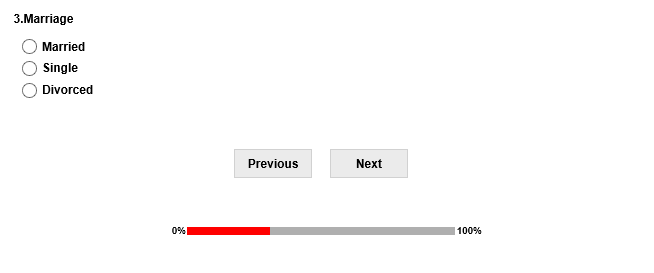


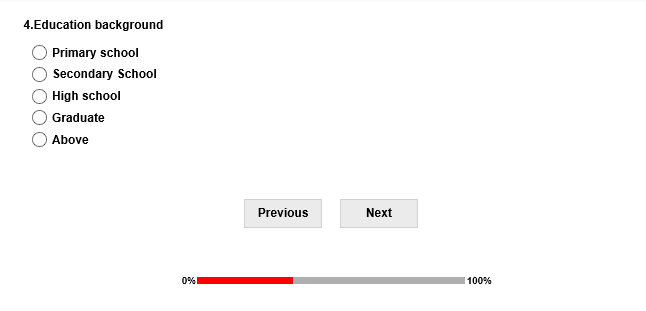


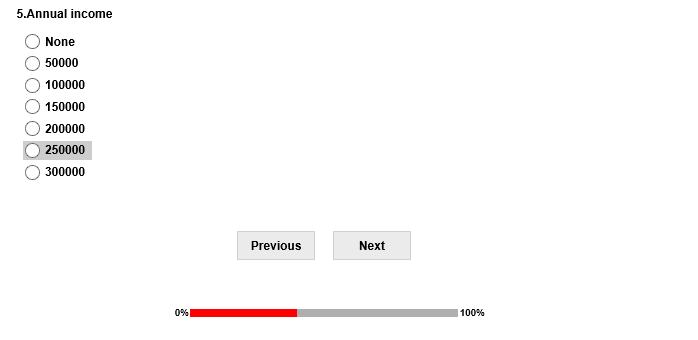


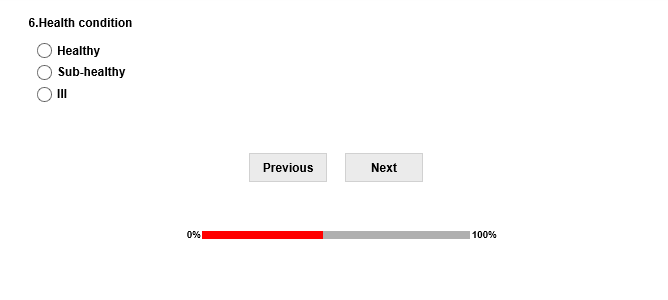


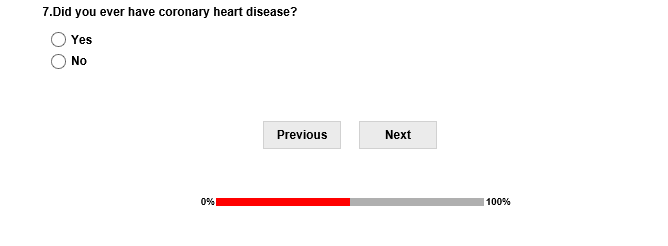


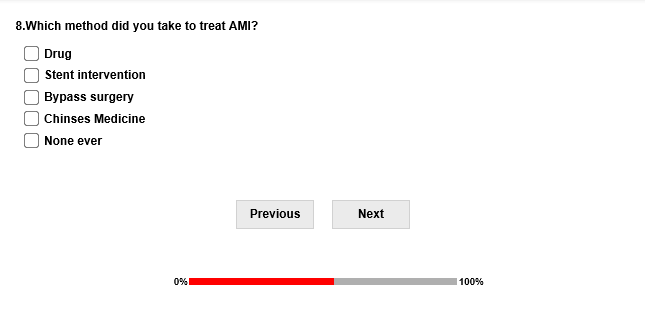


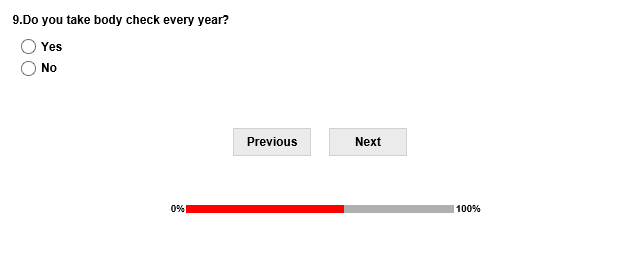


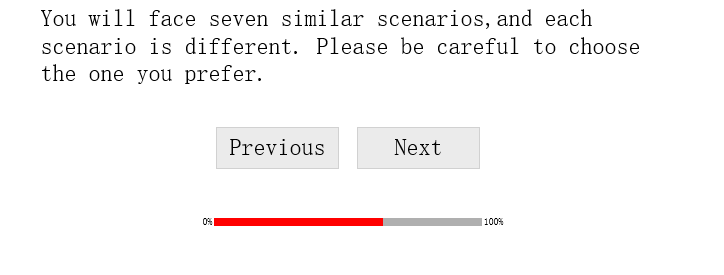


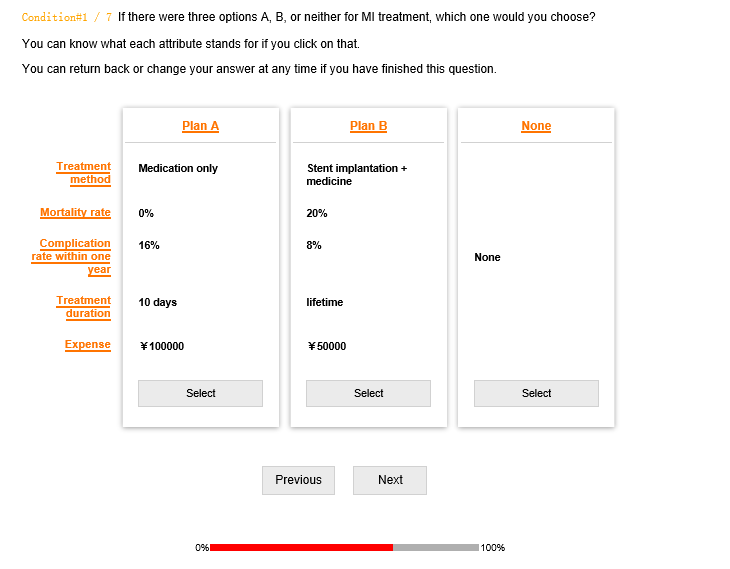

Supplement: Supplementary file 3 — Additional file 3. The website of the questionnaire: http://t.cn/ROlvKtL. [file 12955_2020_1466_MOESM3_ESM.docx]
