# Supplementary material for: Preference of individuals in the treatment strategies of acute myocardial infarction in China: a discrete choice experiment
Source: Health Qual Life Outcomes. 2020 Jul 7;18:217. doi: 10.1186/s12955-020-01466-1 (PMC7339539; doi:10.1186/s12955-020-01466-1)
Supplement: Supplementary file 5 — Additional file 5: Table 2. Demographics and Characteristics of Patients in this Study. [file 12955_2020_1466_MOESM5_ESM.docx]

**Additional File 5 ------ Table 2 Demographics and Characteristics of Patients in this Study**

| **Variables** | **N=383** |
| --- | --- |
| **Sex** |  |
| Male (n) | 164 |
| Female (n) | 219 |
| **Age, mean years** | 30. 4 |
| **Married(%)** | 23.4% |
| **Education** |  |
| Below graduate (%) | 26 |
| Graduate and above (%) | 74 |
| **Annual income, more than RMB$200,000(%)** | 31.5 |
| **Whether did you or your family members have any heart diseases in the past?** |  |
| Yes (%) | 62.6 |
| No (%) | 37.4 |
| **Did you and your family members take a body check every year?** |  |
| Yes (%) | 35.6 |
| No (%) | 64.4 |
