# Supplementary material for: Preference of individuals in the treatment strategies of acute myocardial infarction in China: a discrete choice experiment
Source: Health Qual Life Outcomes. 2020 Jul 7;18:217. doi: 10.1186/s12955-020-01466-1 (PMC7339539; doi:10.1186/s12955-020-01466-1)
Supplement: Supplementary file 6 — Additional file 6. Demographics and characteristics of subgroups in this study. [file 12955_2020_1466_MOESM6_ESM.docx]

**Additional File 6 ------ Demographics and characteristics of subgroups in this study**

|  | | Class 1  n=146 (38.1%) | Class 2  n=177 (46.2%) | Class 3  n=60 (15.7%) | *p value* |
| --- | --- | --- | --- | --- | --- |
| Sex | Male | 52(35.6%) | 71(40.1%) | 25(41.7%) | 0.620 |
|  | Female | 94(64.4%) | 106(59.9%) | 35(58.3%) |  |
| Age, mean (SD) | | 24.6 | 23.4 | 34.3 | <0.001 |
| Has history of marriage | | 35 (24.0%) | 27 (15.3%) | 38 (63.3%) | <0.001 |
| Education (graduate and above) | | 103 (70.5%) | 135 (76.3%) | 27 (45.0%) | <0.001 |
| Income (more than RMB$50,000 per year) | | 43 (29.5%) | 42 (23.7%) | 33(55.0%) | <0.001 |
| History of cardiovascular diseases | | 2 (1.4%) | 3 (1.7%) | 4 (6.7%) | 0.76 |
| Health conditions( healthy) | | 82 (56.2%) | 93 (52.5%) | 38 (63.3%) | 0.215 |
| Have body-check every year | | 63 (43.2%) | 79 (44.6%) | 34 (56.7%) | 0.186 |
